# Building a bridge—an archeologist's perspective on the evolution of causal cognition

**DOI:** 10.3389/fpsyg.2014.01472

**Published:** 2014-12-17

**Authors:** Miriam N. Haidle

**Affiliations:** ^1^Research Center “The Role of Culture in Early Expansions of Humans” of the Heidelberg Academy of Sciences and Humanities, Senckenberg Research InstituteFrankfurt, Germany; ^2^Ältere Urgeschichte und Quartärökologie, Institut für Ur- und Frühgeschichte und Archäologie des Mittelalters, Eberhard Karls UniversityTübingen, Germany

**Keywords:** causal cognition, evolution, tool behavior, problem-solution distance, cognigram, effective chain, cultural performance

## Abstract

The cognitive capacities of fossil humans cannot be studied directly. Taking the evolution of causal cognition as an example this article demonstrates the use of bridging arguments from archeological finds as starting point via identification/classification, behavioral reconstructions, and cognitive interpretations to psychological models. Generally, tool use is linked to some causal understanding/agent construal as the tool broadens the subject's specific capabilities by adding new characters to its action sphere. In human evolution, the distance between the primarily perceived problem and the solution satisfying this need increased markedly: from simple causal relations to effective chaining in secondary/modular tool use, and further to the use of composite tools, complementary tool sets and notional tools. This article describes the evolution of human tool behavior from the perspective of problem-solution-distance and discusses the implications for a linked development of causal cognition.

There are no data available about past human cognition. But if you want to learn something about the causal cognition of past human populations and its evolution you can look for past behavioral evidence. However, there are no direct data available about past human behavior. If you want to learn something about what people did, which knowledge and skills they had and which decisions they made, you should examine the material remains of the past behavior. This is what archeologists are dealing with. The archeological record represents materialized aspects of behavior. However, also within its limits this narrow record is not comprehensive. It is restricted by processes of embedding in the soil, by preservation over thousands or even millions of years, by discovery and the recognition of the significance, by the way of documentation of the find itself and of its context. If everything went well, you still look only at a piece of stone or wood with traces of manipulation in association with other such objects. The object does not speak for itself. Archeologists try to give the artifacts voices through interpretations, which depend on the incorporated knowledge about similar finds, analogies and/or differential diagnoses and their context, but also on current scientific paradigms and on individual experiences and world views. Sometimes the interpretations can or could be falsified, but as long as adequate evidence is lacking the quality of an interpretation relies on the simplicity of the argument and its plausibility in scientific standards. This is a possible starting point from which to explore the evolution of causal cognition. What archeologists can contribute in detail to bridge the gap between material remains of past human behavior and insights in the cognitive background will be discussed in the following explanation.

## Building a bridge—structural design

**Figure 1:**
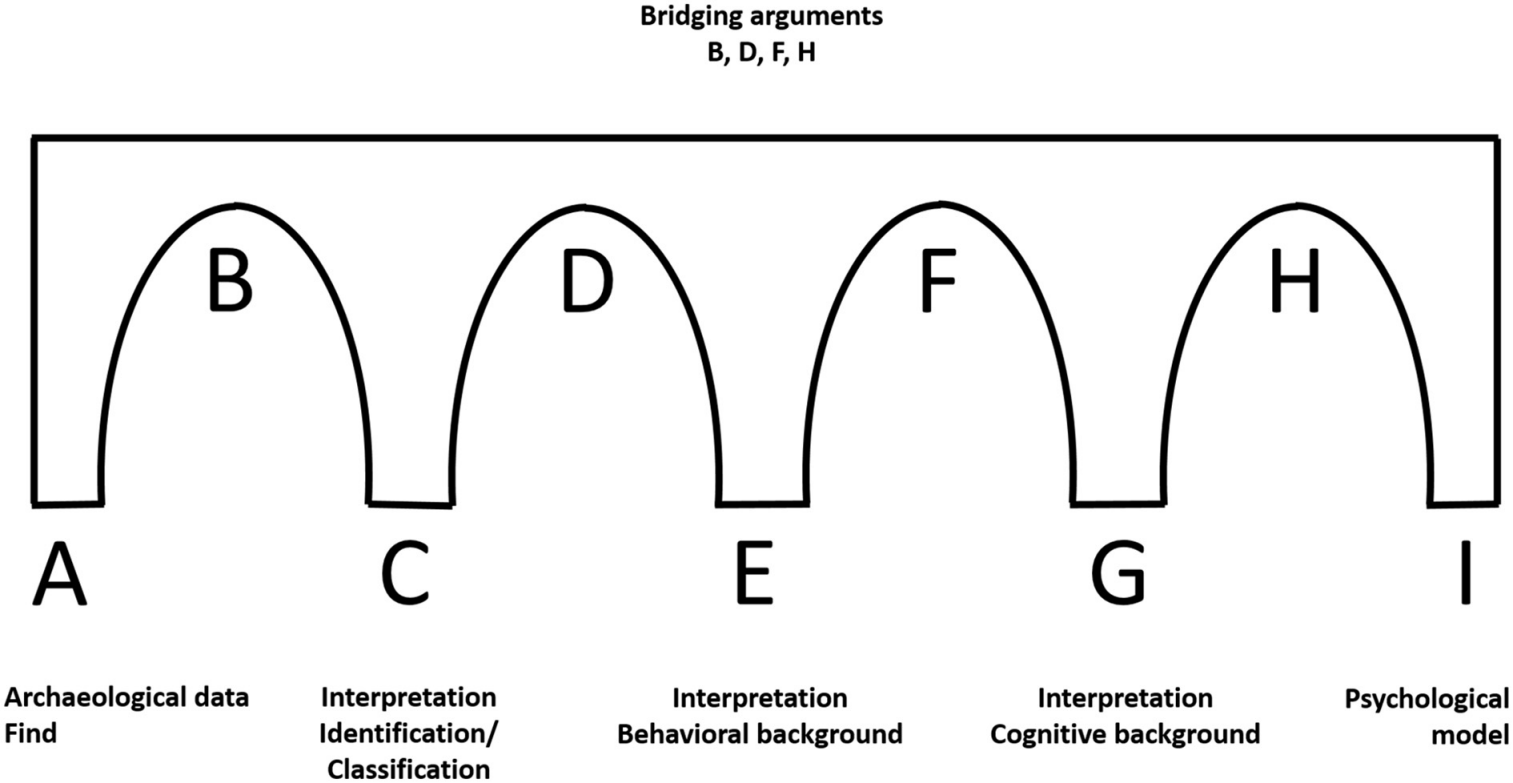
**Bridging arguments: from archeological data to psychological models**.

As explained above, the archeological record does not provide direct insight into the behavior of past hominins, just as artifacts do not give direct evidence on the (causal) cognition underlying the material behavior. Archeological assessments of prehistoric cognition must rest on a series of bridging arguments (Wynn, 2009; Botha, 2010; Wadley, 2013) (Figure 1). For example, a fragmented piece of stone with traces of modification (data A) represents the starting point, the “safe bank.” This object can be identified as part of a composite spear (interpretation C) using artifact attributes such as metric dimensions, weight, and functional interpretations based on the manageability for different purposes, traces of possible use and recent analogies (bridging arguments B). Assuming that the bridge (A–B–C) is correct, we can infer the activities needed to produce such a composite spear the stone point was a part of, how the activities were organized and what artisans had to know, conceive and do, to accomplish their goals (interpretation E). This interpretation is developed with the help of technological evidence, experiments and, again, ethnographic analogies (bridging arguments D). Assuming that the bridges (A–B–C–D–E) are correct, a third group of bridging arguments (F) about the cognitive systems underpinning the activity can then lead to cognitive interpretations (G). These are linked by further bridging arguments (H) to psychological models (I). Tools don't speak for themselves, but have to be interpreted with theories of behavior. The resulting interpretations are pillars from which, with the help of explicit theories of cognition, the platform of probable cognitive requirements of the past behavior can be reached (Garofoli and Haidle, 2014).

## Building the bridge—raw material

The data on which the bridging arguments concerning the evolution of causal cognition rest are tools manufactured and used by animals today as well as by past and recent hominins. Tools are defined here “as freely movable objects that are used in a controlled manner with hands, feet, beaks, mouths, trunks, and tails as an extension of these in order to change the form, position, or condition of another object, organism, or the user himself” (Haidle, 2012, pp. 147–148). Because of their extra-corporal and as such, object status and general materiality tools represent a perfect raw material through which to explore past human behavior. They are materialized products of behavior, have been documented in numerous animal species, mainly in birds and mammals and especially in primates (Beck, 1980), and form the majority of the archeological record.

But tools are also behavioral media; they are deployed in situations in which the subject's capabilities are insufficient or inadequate to cause an effect–that is to change the status (form, position, condition) of the subject itself or another object. The subject operates the tool as causal *agens* with the implicit intention that it causes an effect. Although it is the subject that initiates and controls the action of the tool, it is the tool and its specific qualities that produce a change in form, position, or condition of the target; therefore, and in this context, the tool is regarded as an agent with active potential. A chimpanzee opens a nut with a hammerstone (Boesch and Boesch, 1984): the animal handles an *agens* that she selected from the environment and that possesses qualities making it more capable than herself to solve her problem. A New Caledonian crow uses a modified twig to extract insects from holes in dead wood (Hunt, 1996): the *agens*–chosen, modified and manipulated by the animal–has a specific effect on the desired object; it causes a change of status of the prey. Tool behavior deals with this form of agents/*agens* and effects, and thus it is a perfect starting point to examine the unfolding of causality-based behavior in human evolution in comparison to the faculties of recent animals.

Additionally, tool behavior allows the search for a cognitive background (cf. McCormack et al., 2011). There are few examples of tool use in animal behavior which are probably triggered mainly or exclusively by instincts such as the use of a hammering device by wasps of the genus *Ammophila* and *Sphex* to close their breeding cavern, or ant lions throwing sand to let prey slide into a sand pit (Beck, 1980). Most cases of tool behavior seem to be more or less selective and flexible (cf. Seed and Byrne, 2010). Although often an inborn tendency to manipulate objects can be observed in tool-using species, the specific tool behaviors are acquired in an individual or social learning process not only in how a tool is applied, but also why this item serves as a tool to solve a problem better than another item. Causal reasoning as the ability to identify the relationship between causes (in tool behavior: tools as agents/*agens*) and effects (the change of the status of an object on which the tool is applied) is fundamental to conceptualize tool use. Goswami and Brown state that: “··· the conceptual structure may be heavily dependent on causal relations, with natural concepts always needing to be embedded in causal theories to have real meaning or inductive power” (Goswami and Brown, 1989, p. 70; see also Keil, 2006). To obtain the meaning as hammer a stone needs to be embedded in causal theories about hard and heavy items and their potential effect to open nuts. If the hammer stone solution is not only used in one specific problem-solution setting, but also transferred to other problems than nut-cracking, even a broader causal theory (and analogical reasoning) is necessary. And the causal theory has to be extended further in the chaining of several tools and their effects as it is typical for human tool behavior. *Homo heidelbergensis* produced and applied different stone tools to shape a wooden thrusting spear to hunt for horses: 300,000 years ago humans made heavy-duty tools and sharp flake tools and used them to fell small trees; remove the bark, branches and twigs; optimize the form (possibly also with the help of fire and water); and smooth the surface in a process probably lasting several days (Thieme, 1997; Haidle, 2010). The process of transforming a small tree into a hunting gear with the help of different tools depends on causal understanding–that is the development of a functional theory about physical properties of raw materials and tools and the mechanisms that change the status of the target. Besides applying different agents/*agens* in a chain of effects in order to receive a dietary income, the human being had to control the impulses, inhibit spontaneous reactions, learn individually as well as in social and historical contexts, and plan the activities to gain a delayed profit. The manufacture and use of tools are determined by several cognitive aspects, including different levels of causal reasoning, and are commonly reproduced culturally. As such elements of behavior, tools are well suited to build the bridge to reach into the blackbox of past human cognition.

## Building the bridge—construction work

Archeologists have to interpret their raw data, the archeological finds, with the help of bridging arguments (A–B–C) to proceed in further steps with the reconstruction of the activities, knowledge, and conceptions behind the manufacture and use of the tools (C–D–E). The studies of animal tool behavior begin at a different point as most of the raw data stem from direct, though often fragmented or anecdotic observations of an animal's practice with a certain tool. Ethologists mainly start at C, and the bridge (C–D–E) has to be reconstructed only partially. To parallelize the bridges of different archeological artifacts and of animal tool behavior the data have to be made comparable. To represent the individual bridges in a contrastable way the underlying perceptions and behavior in the process of manufacture and use can be coded in cognigrams and effective chains (see e.g., Haidle, 2010, 2012; Lombard and Haidle, 2012; Hunt et al., 2013). This method is based on the problem-solution distance approach, which originates in the comparative research of Wolfgang Köhler (1926) and takes each tool behavior as an extension of a simple and direct way from need to satisfaction. While a hungry sheep has only to bend the neck to feed on grass, a chimpanzee with appetite for termites has to find or produce an appropriate probe to extract the insects from their mount to appease her hunger. The use of a tool incorporates a moment of inhibition of the impulse to satisfy a need as quickly as possible; the distance between a problem and its solution is increased.

**Figure 2:**
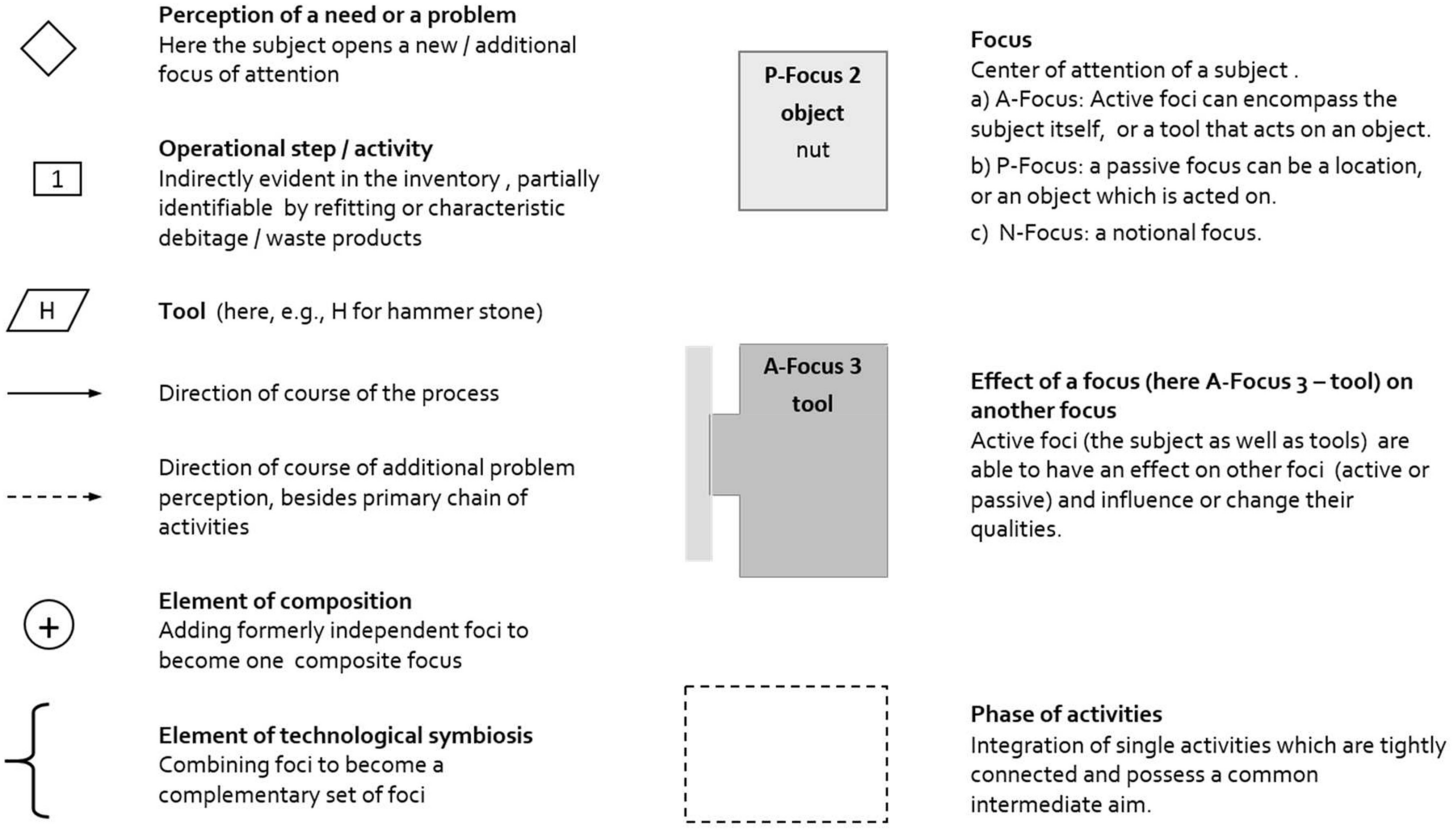
**Graphic elements of a cognigram**.

**Figure 3:**
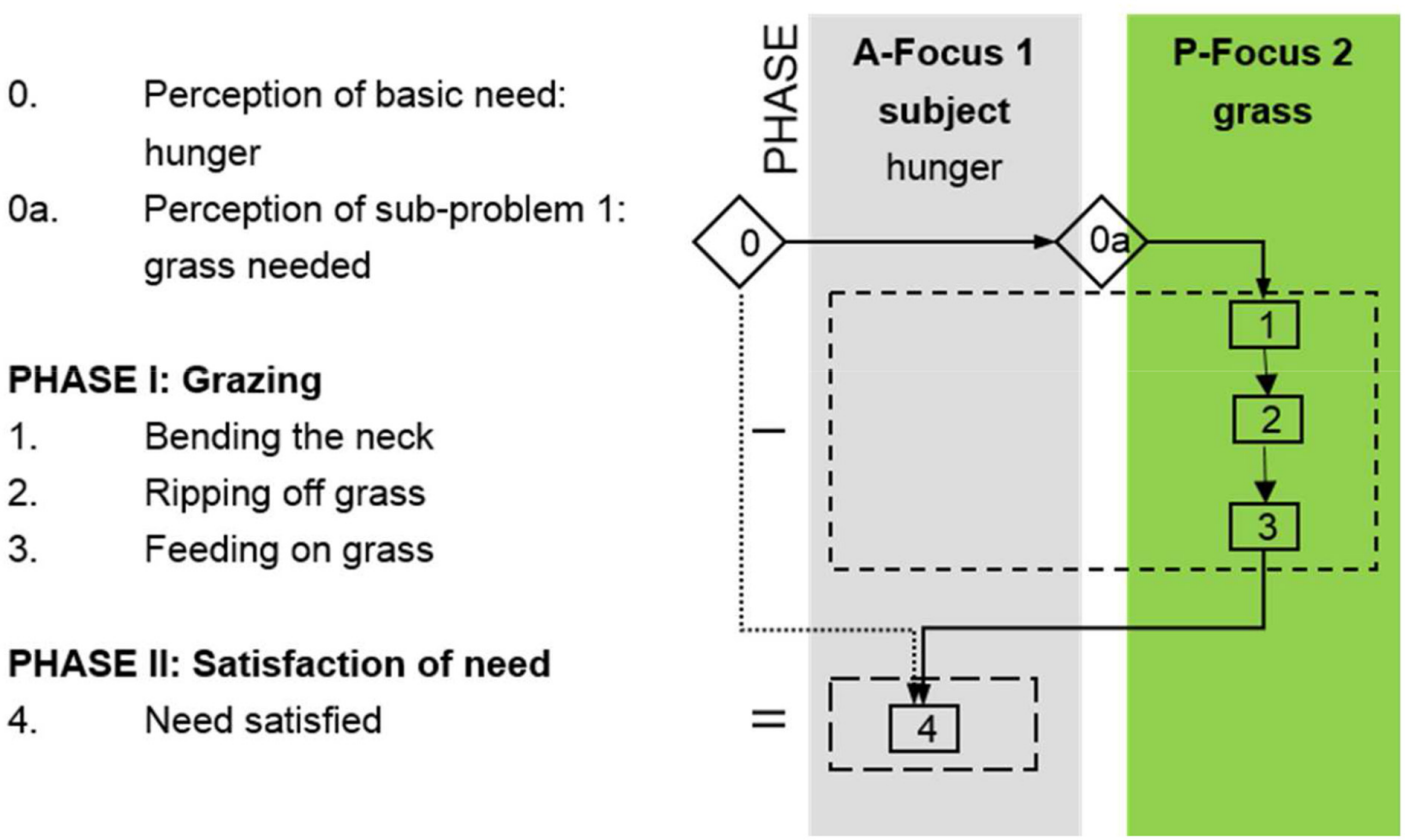
**Cognigram depicting the problem-solving behavior of a sheep grazing to satisfy the basic need “hunger**.”

**Figure 4:**
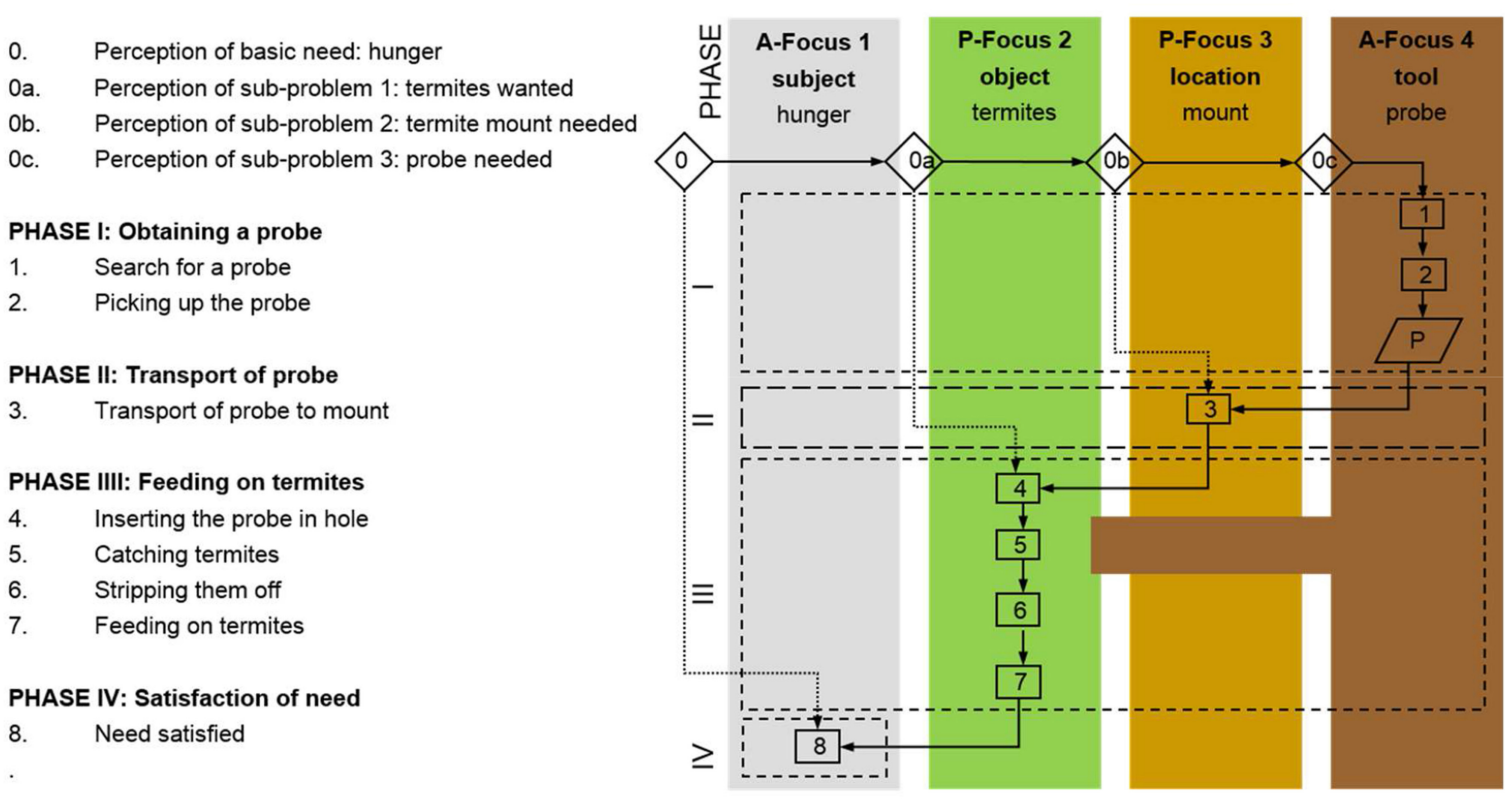
**Cognigram depicting the problem-solving behavior of a chimpanzee using a probe to feed on termites to satisfy the basic need “hunger**.”

The extension of the perception of a need and the following actions can be systematically coded and illustrated in cognigrams (Figure 2). Starting with the subject's perception of a basic need, a line of subsequent problems is perceived, opening new attention foci, which are acted upon to satisfy the basic need. The attention foci can be classified as active if they are actively controlled by the subject and act upon other foci. They can encompass the subject itself or the tools. In contrast, passive foci are objects that are acted upon or locations. Returning to the examples of the sheep and the chimpanzee the method becomes clear. The sheep (subject) feels hungry (basic need, first attention focus) and wants to eat some grass (second attention focus), bends the neck (action 1) to rip off the grass (action 2) to feed on (action 3) to become full (satisfaction of need) (Figure 3). While the grazing-sheep example describes a basic problem-solution distance with the subject as the only agent, the grass as the object and bending the neck and grazing as necessary actions, tool behavior always represents an extension of the problem-solution distance with at least one more active attention focus (the tool) with a certain effect. If the chimpanzee (subject) feels hungry (basic need, first attention focus) and wants to feed on termites (second attention focus), the animal looks for an adequate location (third attention focus), perceives the additional need of a probe (fourth attention focus), which has to be searched for (action 1), obtained (action 2), and transported to the termite mount (action 3), to insert it into holes of the mount (action 4) to catch the insects (action 5), which cling to the probe (effect of tool), to strip them off the tool (action 6), and to feed on (action 7) to become full (satisfaction of need) (Figure 4).

**Figure 5:**
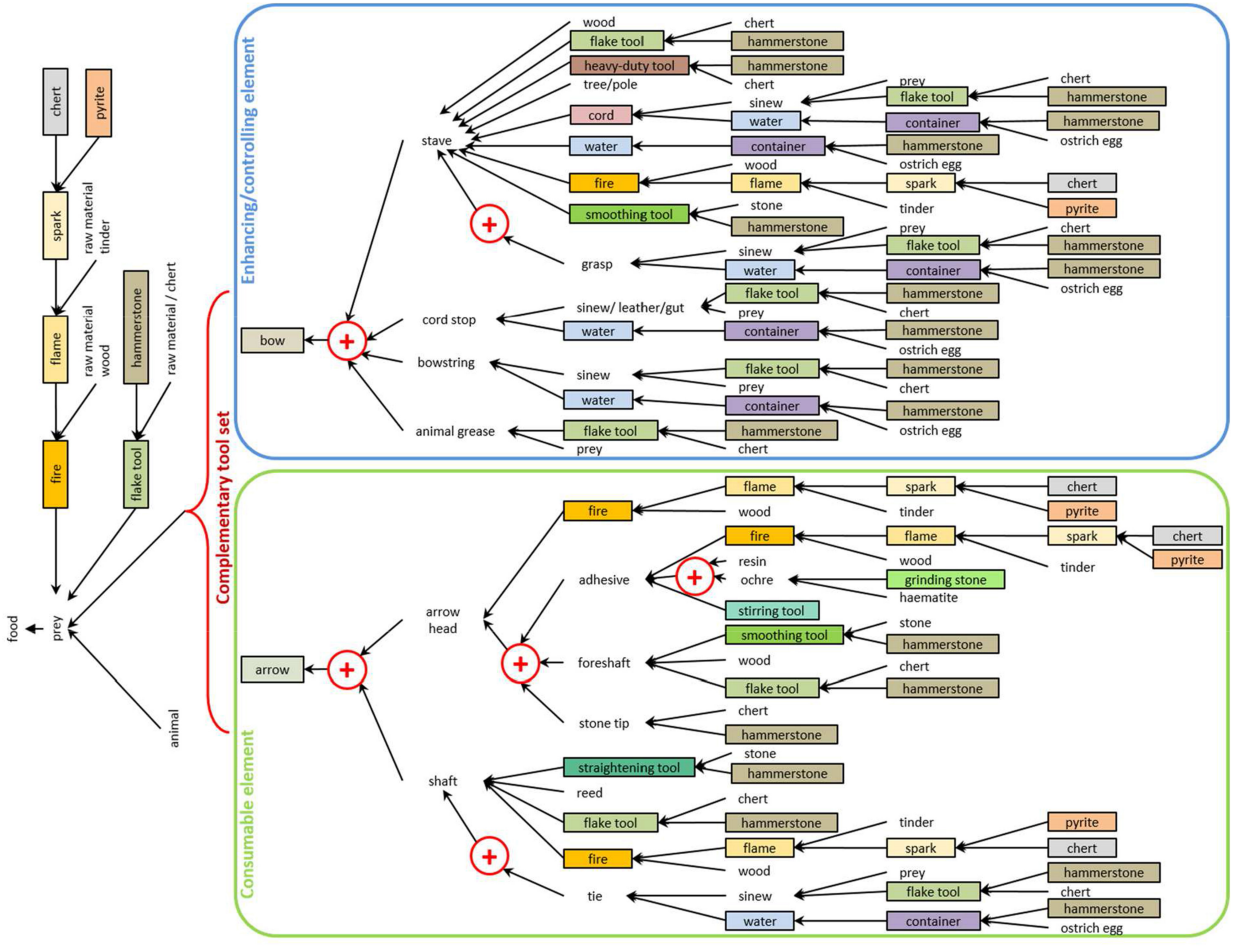
**Simplifying a complex set of behaviors**. The effective chain depicts agents (tools in boxes, raw materials and intermediate products without boxes) and effects (arrows, plus signs, and curly braces) and summarizes the combination of more than two dozen behavioral modules in the manufacture and use of a bow-and-arrow complementary tool set (from Lombard and Haidle, 2012).

In cognigrams, the different elements of a behavior are broken down by active and passive attention foci (subject, tools, objects, locations), by perceptions of need opening the attention foci, by actions within or directed to an attention focus, by effects of attention foci on other attention foci, and by phases–clusters of actions that have to be executed as a group or, if interrupted, started again with the first action of the phase. A crucial point for the comparison of behaviors is an equivalent starting point (basic need) and the tracking of all elements including actual or probable interruptions until the final satisfaction of the basic need. The cracking of nuts with a hammerstone by chimpanzees is not directly comparable with the production of a simple stone tool with a hammerstone by a hominin, because the manufacture of the stone tool is only part of a process to fulfill a basic need, which can be the satisfaction of hunger or defense, for example (cf. Haidle, 2010). If tool behavior includes several tools with different effects to fulfill a need, the cognigrams can be simplified to effective chains that represent only the attention foci of the behavior and the effects they have on each other (Figure 5) (Lombard and Haidle, 2012). Cognigrams and effective chains, however, are only as good as the reconstructions of the behavior they illustrate. Cognigrams therefore consist a) of a formalized description of the reconstruction of the behavior with the elements in chronological order of appearance and b) of a graphical representation. The bridging arguments (D) that lead to the interpretation of the behavioral background (E) shown in the cognigrams are given in a reference section explaining the background and listing the sources.

The reconstruction of the behavioral elements contributing to the overall picture of a certain problem-solution unit can be more or less detailed and can vary. Even descriptions of direct observations of a problem-solution unit can identify different details, e.g., in problem perceptions and actions, and variegate them by splitting or lumping; the more so do reconstructions based on archeological finds. The following options A, B, and C of the grazing-sheep case exemplify how different the descriptions can be even in such a simple instance.

Option A

1. First attention focus, basic need: the sheep (subject) feels hungry
2. Second attention focus: the sheep identifies edible plants (object)
3. Action 1: the sheep bends the neck···
4. Action 2: rips off the plants···
5. Action 3: feeds on them ···
6. Action 4: and becomes full (satisfaction of need).

Option B (shortened version)

1. First attention focus, basic need: the sheep (subject) feels hungry
2. Second attention focus: the sheep identifies edible plants (object)
3. Action 1: feeds on them ···
4. Action 2: and becomes full (satisfaction of need).

Option C (extended version)

1. First attention focus, basic need: the sheep (subject) Subfocus A (referring to subject): notices that the stomach feels strange/hurts
  Subfocus B (referring to subject): “realizes” that it is hungry
  Subfocus C (referring to subject): “knows” that it needs to eat something
2. Second attention focus: the sheep identifies edible plants (object)
3. Action 1: bends the neck close to the grass ···
4. Action 2: opens the mouth ···
5. Action 3: rips off the grass ···
6. Action 4: chews the grass ···
7. Action 5: tastes whether it is good or not ···
8. Action 6: swallows the grass ···
9. Action 7: and becomes full.
10. Re-opening of first attention focus, satisfaction of need: the sheep (subject)
  Subfocus A (referring to subject): notices that the stomach feels better
  Subfocus B (referring to subject): “realizes” that the hunger is gone
  Subfocus C (referring to subject): “knows” that it can stop feeding

Although the grazing-sheep case shows at first sight impressive differences in depiction, the lumping and splitting of subfoci/main foci and of operational steps/actions do not really change the overall picture of main active and passive foci and their effects on one another. If, however, new elements are added or old ones are completely omitted (instead of being separated from or integrated in more comprehensive steps), then real variants of a problem-solution unit are documented. Commonly, the reconstructions of prehistoric behavior (E) and the cognigrams as their graphic representations depict idealized behavioral processes derived from a multitude of slightly different possibilities. To give a current example: several observations of brewing coffee with hot water and a simple paper filter lead to a generalized description of the behavioral process; the planning differences about the facility used to boil water, whether coffee beans are first ground in a mill or ready-made powder is used, and the amount of coffee powder taken are not discussed in detail. It depends on the aim of the analysis if this idealized description is sufficient. The idealized depiction is sufficient, if you want to compare traditional German coffee making with simple paper filter with an Ethiopian coffee ceremony or with the use of a coffee dispenser. It is not sufficient, if you want to study variability in the behavior of an individual, small differences within or between groups, or changes in family traditions of the same behavior “brewing coffee with a simple paper filter.” For the identification of major leaps in behavioral concepts in human evolution major changes in the reconstructed behavioral processes have to be identified. The fundamental reconstructions (E) have to be evaluated regarding the preceding argumentative bridge (A–B–C–D).

The possibility of equifinality, the fact that a problem may be solved by different means, that a tool may be manufactured and applied in different ways, raises the question of how convincing the reconstructions (E) and their graphic representations in cognigrams are. To avoid the possibility of equifinalities, or to discuss the alternative ways of problem-solution in-depth, the underlying argumentative bridge (D) has to be given special consideration. Therefore, technological evidence on the artifact such as traces of manufacture and use wear, together with data obtained from experiments or ethnographic analogies have to be thoroughly described. And it has to be discussed (a) to what extend especially simpler alternatives of behavioral processes could produce similar results, (b) if elements, on which an identification of a leap is based, are really necessary, and (c) if the contextual evidence points to the possible or probable parallel application of different ways of solving a problem. Nevertheless, even the most thorough reconstruction process only remains valid until it is replaced by a simpler explanation or a hypothesis that comprises more evidence. Equifinality is a problem immanent to all reconstruction processes; and sometimes no decision for one or the other way of reconstruction can be taken. Cognigrams, however, help to facilitate the discussion about the alternatives in clearly showing the differences of the reconstructed processes.

## Building the bridge—job site

With the help of cognigrams and effective chains with which the bridging (C–D–E) is formalized and illustrated, numerous small bridges from single artifacts or tool types (A) to the interpretation (E) can be constructed and set parallel to each other to form a more load-bearing bridge. If these bridges are set in a chronological order, it creates a historical perspective and the course of development becomes visible. This procedure indeed makes it possible to document the expansion of the causal structure of agents/*agens* and effects that accompany the development of tool behavior in human evolution.

### Simple tool behavior

**Figure 6:**
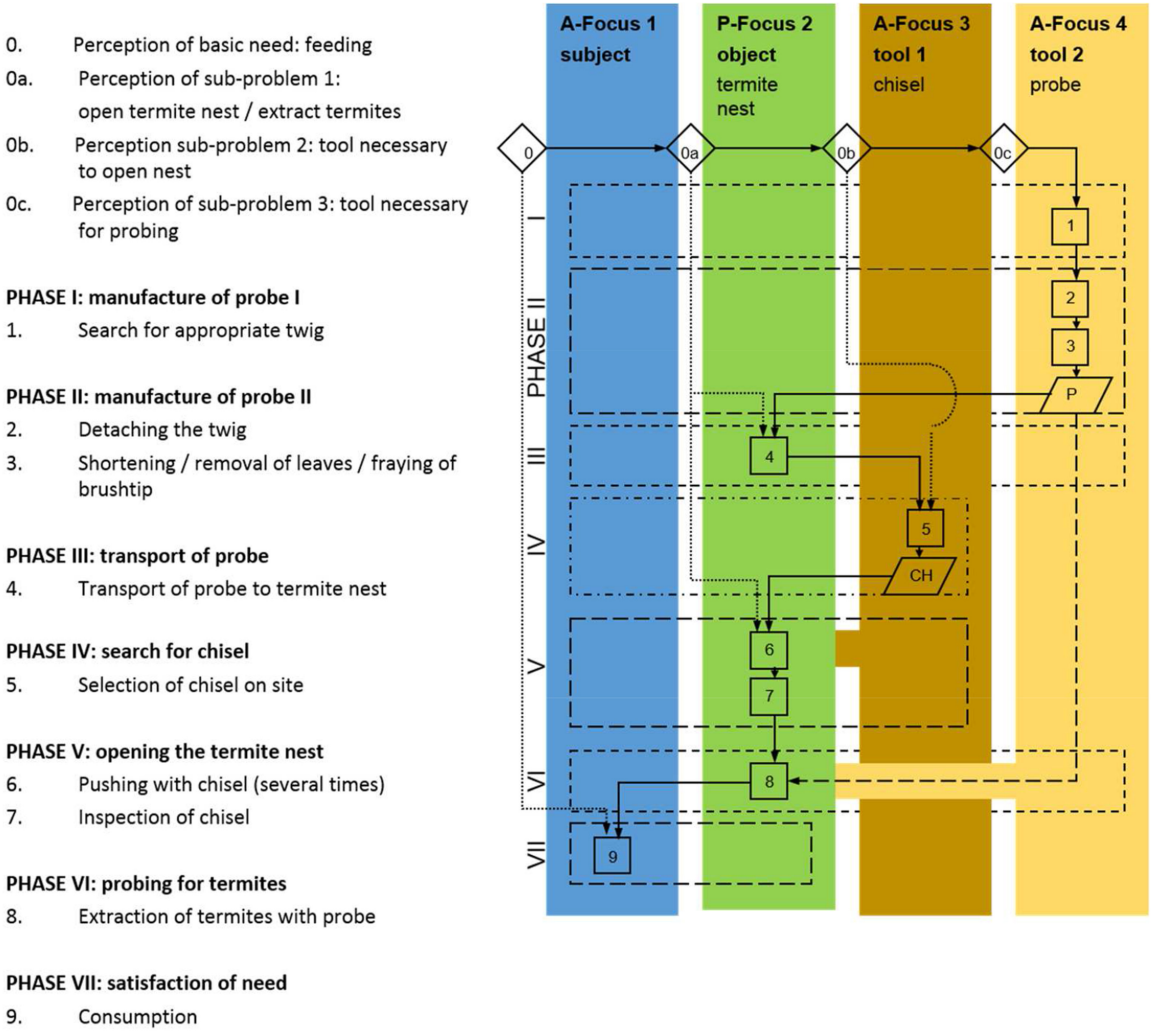
**Cognigram of a chimpanzee using a tool set of chisel and probe to extract termites (after Sanz et al., 2004)**.

Simple tool use comprises the application of one or several tools on one object. The tools can be unmodified or modified with the help of the subject's own facilities (Figure 4; for the variety of simple tool use in animals and the representation of these behaviors in cognigrams see Haidle, 2012). Basis of a selective and flexible tool behavior (cf. Seed and Byrne, 2010) are (a) the inhibition of impulses, (b) a certain perception of an agent-effect or means-end relation that is applied in a tool-on-object behavior (for the discussion of the possible range of perception see below), and (c) a goal-directed manipulation of the chosen tool. Capuchin monkeys, for example, select hammerstones to open nuts according functional features like friability and weight (Visalberghi et al., 2009). Chimpanzees use different tool sets (perforators and probes) to extract termites from subterranean and aboveground nests: they choose the suitable means to get the desired result (opening the different termite nests). In addition, they search for both elements of the tool sets, perforators and probes, in advance before approaching the nests (Sanz et al., 2004) (Figure 6).

### Modular tool behavior

**Figure 7:**
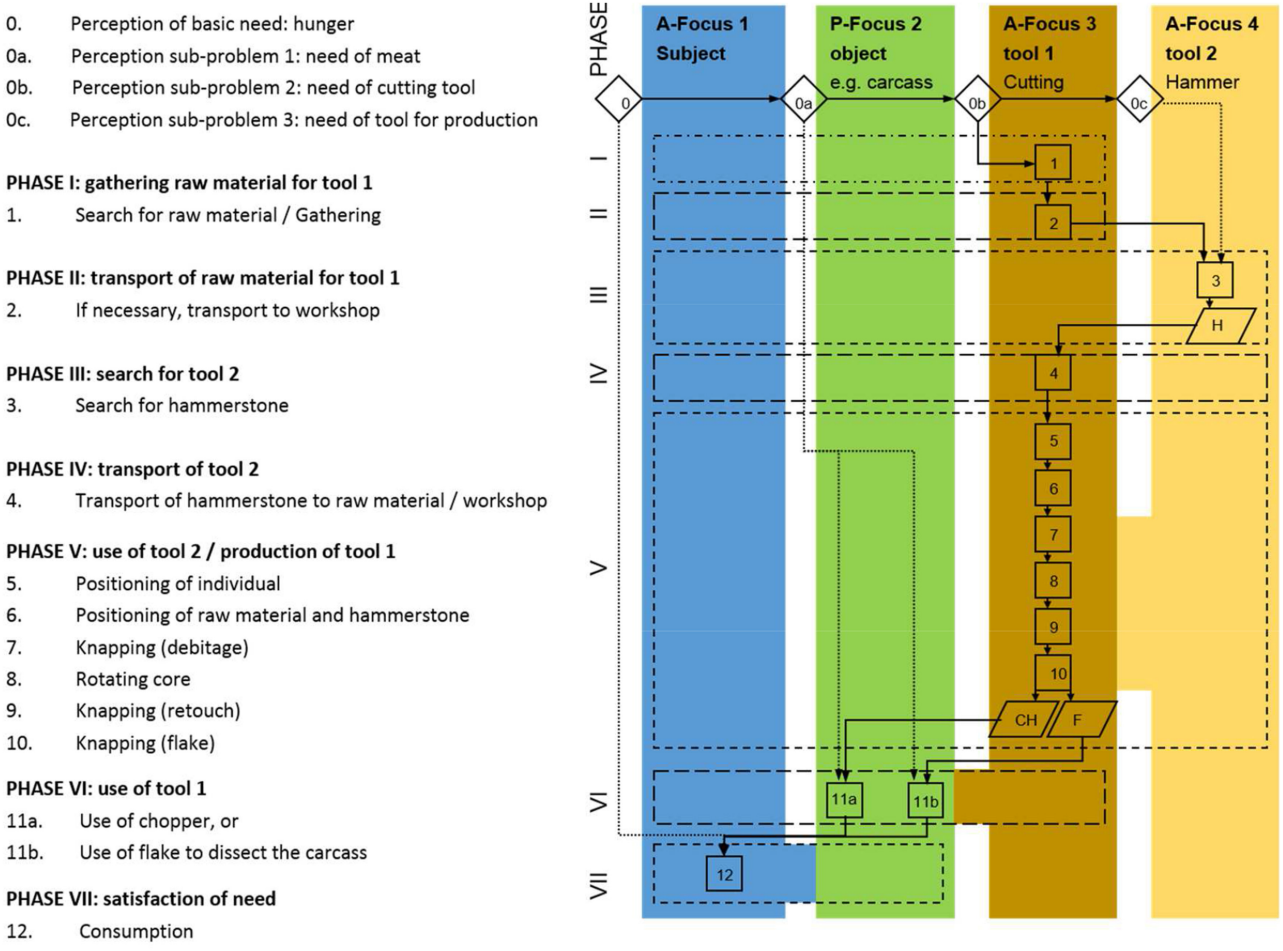
**Cognigram of a *Homo sp*. producing a simple flake or chopping tool with a hammerstone and using it to dissect a carcass**.

**Figure 8:**
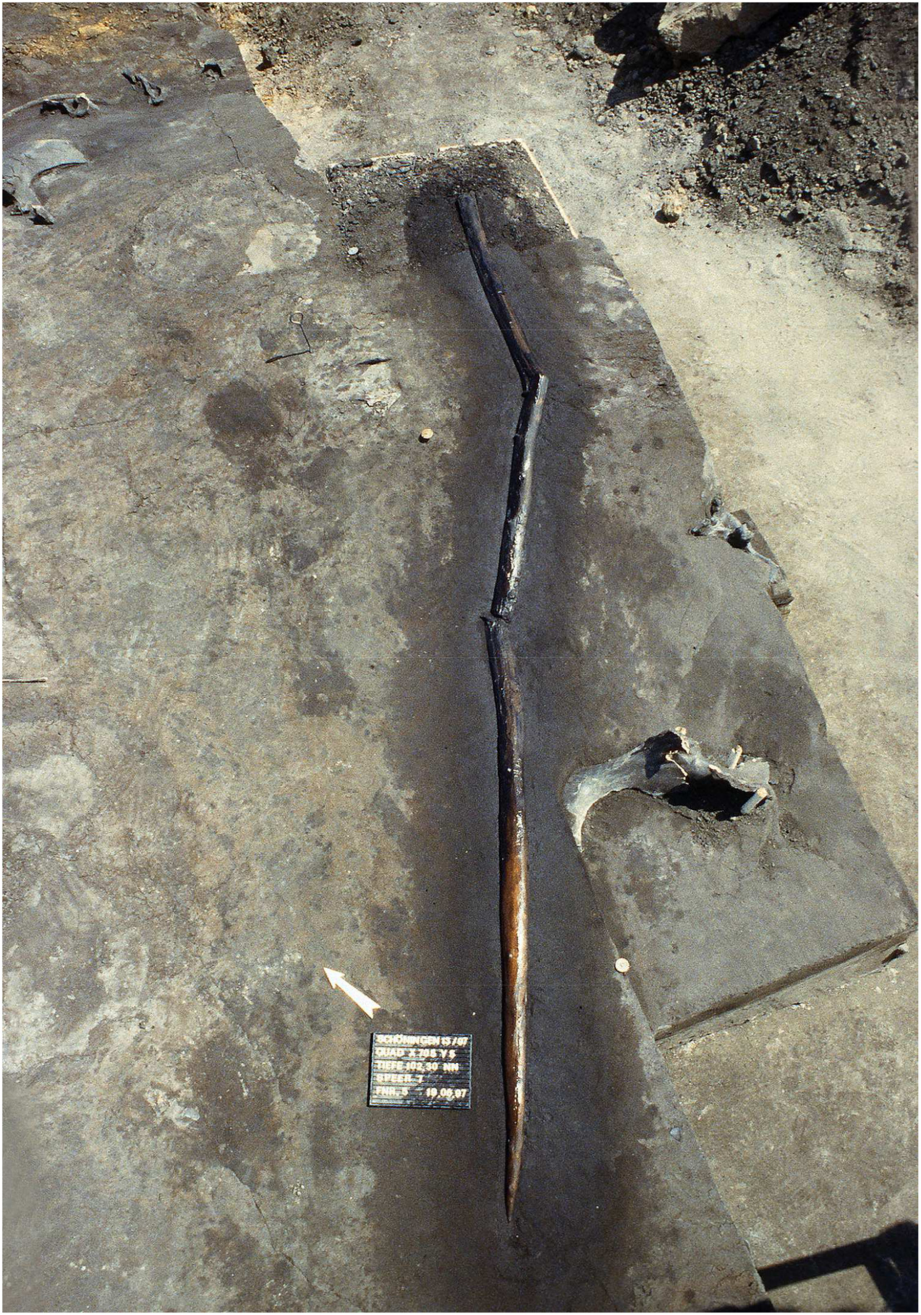
**A sophisticated example of modular tool behavior: a 300,000-year-old wooden spear from Schöningen (Photo: P. Pfarr, Niedersächsisches Landesamt für Denkmalpflege, Wikimedia Commons)**.

An extension of the problem-solution distance beyond the application of a set of several simple tools on one target becomes evident with secondary tool use, the use of tools to produce other tools to solve a problem (Kitahara-Frisch, 1993). Not only intermediate targets in direct connection to the satisfaction of the basic need have to be perceived, but also tools have to be prepared in advance to change the status of an object to become the tool to solve the problem. Such a chaining of agent-effect relations is the foundation for the manufacture of stone tool by hominins reaching back at least 2.6 million years (Semaw et al., 2003): a hammerstone and adequate stone nodules as raw material have to be organized in order to produce cutting tools to process e.g., animal carcasses (Figure 7). So far, the chaining of different agent-effect relations has not been observed in animals in the wild. Experiments with capuchin monkeys imply that this species is able to understand the relationship between two items (tool and food object), but lacks the understanding of the relationship between three items (Fujita et al., 2003), a necessary condition of secondary tool use. Associated with the use of secondary tools is the chunking of parts of the tool behavior into independent behavioral units, which can be combined in different ways to act on and modify one another. A hammerstone can not only be perceived as a means to solve a basic problem like the exploitation of hard food resources, but can also be used to solve secondary problems such as manufacturing of tools. In human evolution, tool behavior becomes increasingly decoupled from basic needs. Behavioral units are not exclusively bound to specific and acute problems. Instead, the elements of behavioral units (stimulus, concept of solution, goal) are increasingly abstracted from specific purposes and become applicable in different contexts: a modular capacity arises. The execution of modular cultural capacities can occur on various technological levels based on differing knowledge and skills: knapping stone tools with different techniques only takes a few minutes, yet requires the same modular cultural capacity as does the manufacture of a simple wooden spear which is likely to span several days (Haidle, 2010) (Figure 8).

### Composite tool behavior

**Figure 9:**
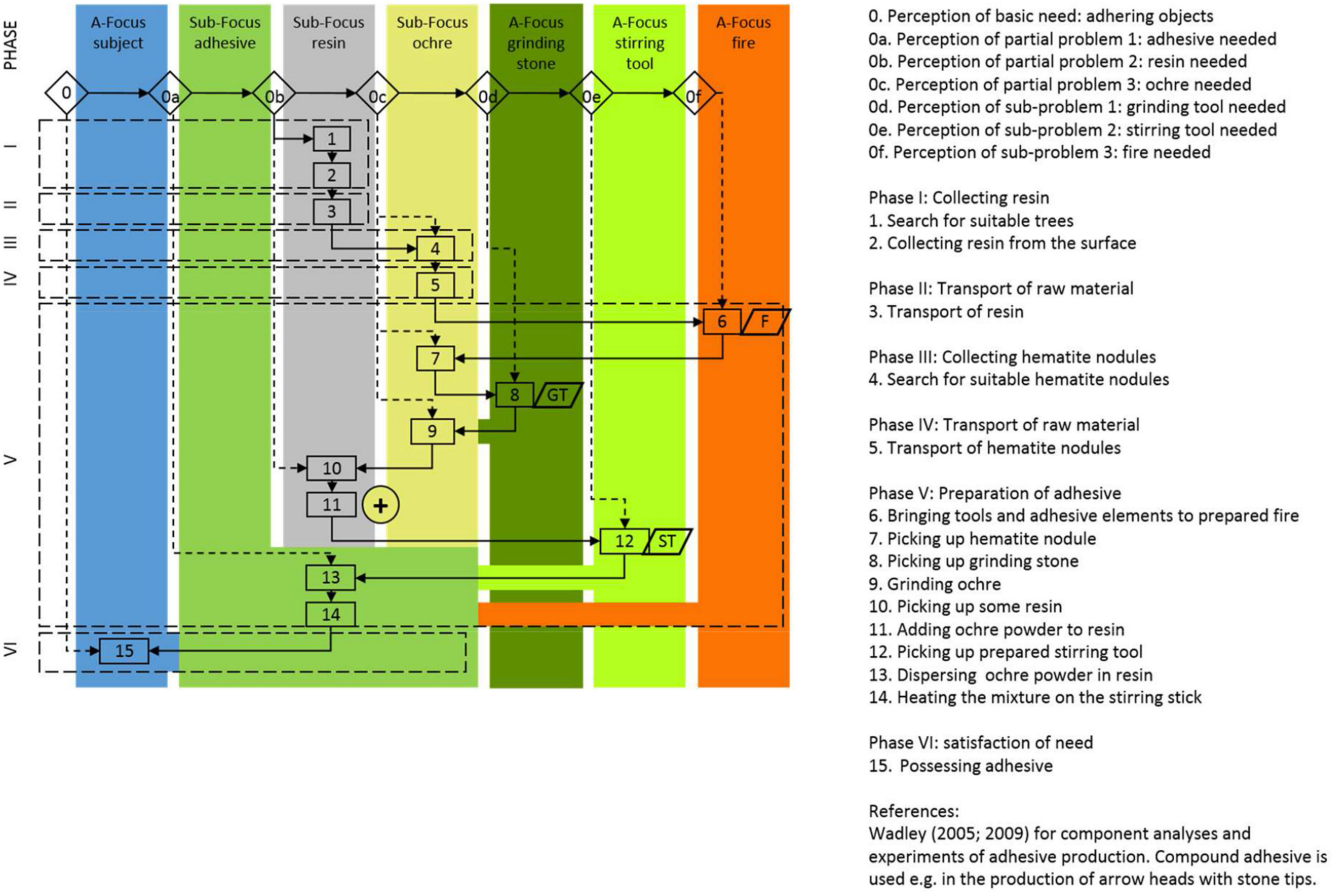
**Cognigram of the production of compound adhesive: note the fusion of resin and ocher (plus sign) becoming a new attention focus “adhesive” (after Wadley, 2005; Wadley et al., 2009)**. The production of compound adhesive is a distinct behavioral module and can be combined with various other modules as in the production of a bow-and-arrow set (see also Figure 5) (from Lombard and Haidle, 2012).

New qualities in the perception of agent-effect relations are the basis of composite tools. In composite tools such as a wooden spear armed with a stone projectile, the problem-solution distance is extended to a combination of different behavioral units with specific qualities (wooden spear with good flight qualities, projectile point made from stone with good cutting properties, adhesive and binding material with good fixing potential) that are fused to form composites with new qualities (composite spear with increased penetrating power). While tools made out of many pieces of the same kind, such as a piles of boxes to be used as a ladder as documented for chimpanzees (cf. Köhler, 1926) or sophisticated baskets made by humans, only escalate the properties of the basic element, composite tools demonstrate a new combination of different qualities. The different elements of a composite tool “may be obtained at different times and in different places” (Ambrose, 2010, S139) while the new functional unit may be assembled much later (Ambrose, 2010). Within the archeological record, hafted tools and compound adhesives (Wadley, 2005; Wadley et al., 2009) are typical material examples of such composites (Figure 9). Early evidence of composite capacity reaches back at least 200,000 years with finds of stone tools with wear traces of wooden hafts in Africa (Rots and Van Peer, 2006) and stone tools from Neanderthal contexts in Italy fixed with birch tar to now decomposed handles (Mazza et al., 2006).

### Complementary tool behavior

**Figure 10:**
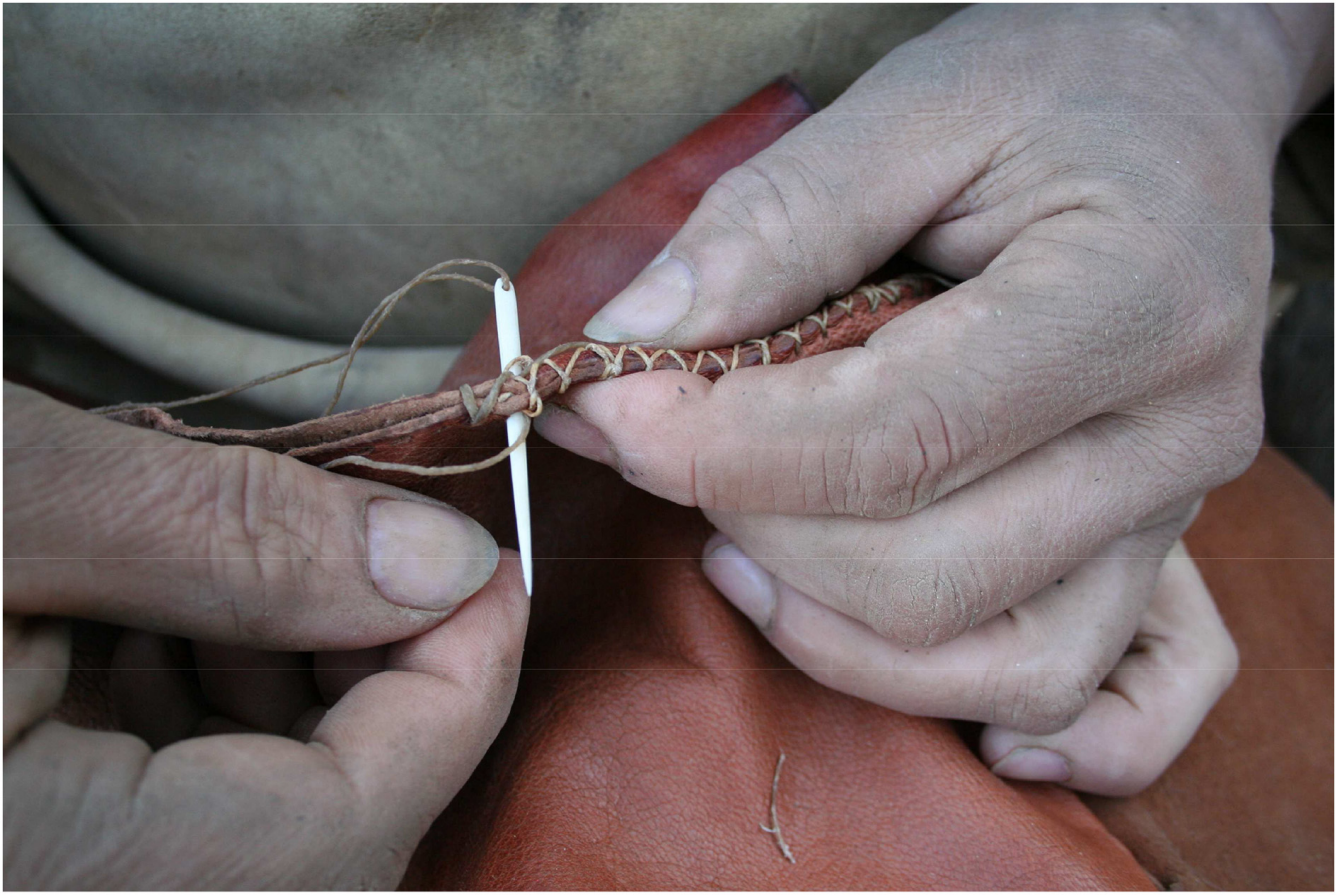
**A complementary tool set: sinew fibers controlled by an eyed-needle made from bone (Photo: Rudi Walter)**.

**Figure 11:**
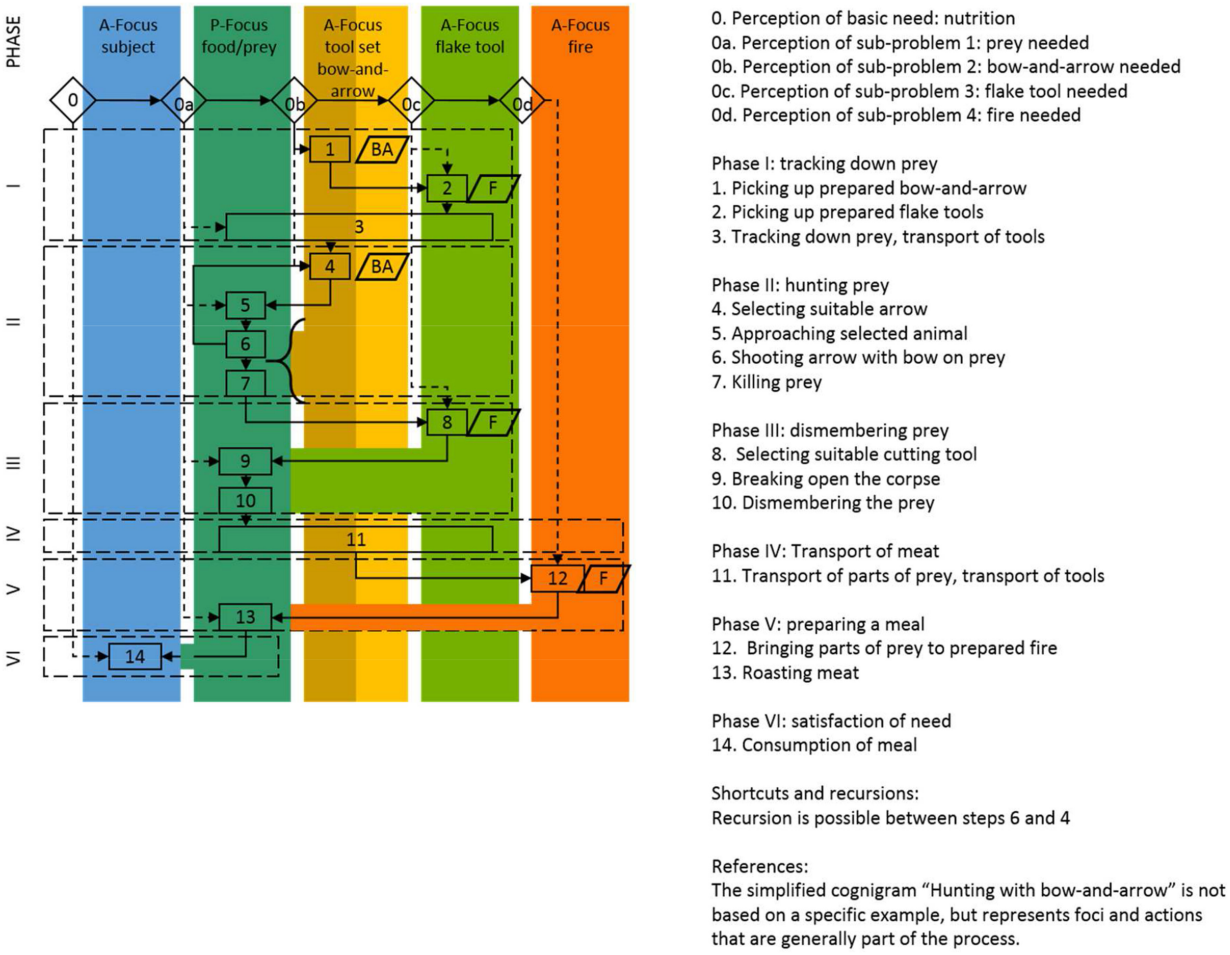
**Cognigram of hunting with a bow-and-arrow-set: note the curly brace indicating technological symbiosis (from Lombard and Haidle, 2012)**. The hunting process may, of course, be an independent module separate from transport, preparation of a meal, and consumption.

While the subject generally operates composite tools, complementary tool sets apply a new aspect of problem-solving with a tool controlling or enhancing another tool which provides the actually desired effect. Bow-and-arrow, needle-and-thread, screw-and-screwdriver, key-and-lock are only some examples of the symbiotic relationship of two discrete, but concerted elements working together to fulfill a common task (Lombard and Haidle, 2012) (Figure 10). Figure 11 shows the cognigram of the application of bow-and-arrow for hunting: note here the curly brace on the effect of the bow-and-arrow set on the prey, indicating technological symbiosis (for a detailed depiction of all behavioral modules necessary for bow-and-arrow manufacture see Lombard and Haidle, 2012; for an overview of foci and effects in the complete process of manufacture and use of a bow-and-arrow see Figure 5). The elements of a complementary tool set must be developed and used as acting entities with two or more interdependent and exchangeable parts in complementary correspondence with each other. To solve a problem with a complementary tool set two different agent-effect relations have to be taken into account, which are released by only one action of the subject: the acting individual draws the bowstring, for example, and lets it go, which propels the arrow, and the arrow consequently penetrates the prey in order to hurt or kill it. The impulse for the goal-directed tool respectively, its effect is given by the controlling/enhancing tool of the complementary set and only indirectly by the subject. As early archeological evidence of complementary behavioral capacities, stone tips from South African sites are discussed, which were probably used as projectile points of arrows and date back to ca. 64,000 years (Lombard, 2011). Eyed needles and parts of spear-throwers are other archeological finds which give hints on the use of complementary tool sets between 30,000 and 10,000 years ago.

### Notional tool behavior

**Figure 12:**
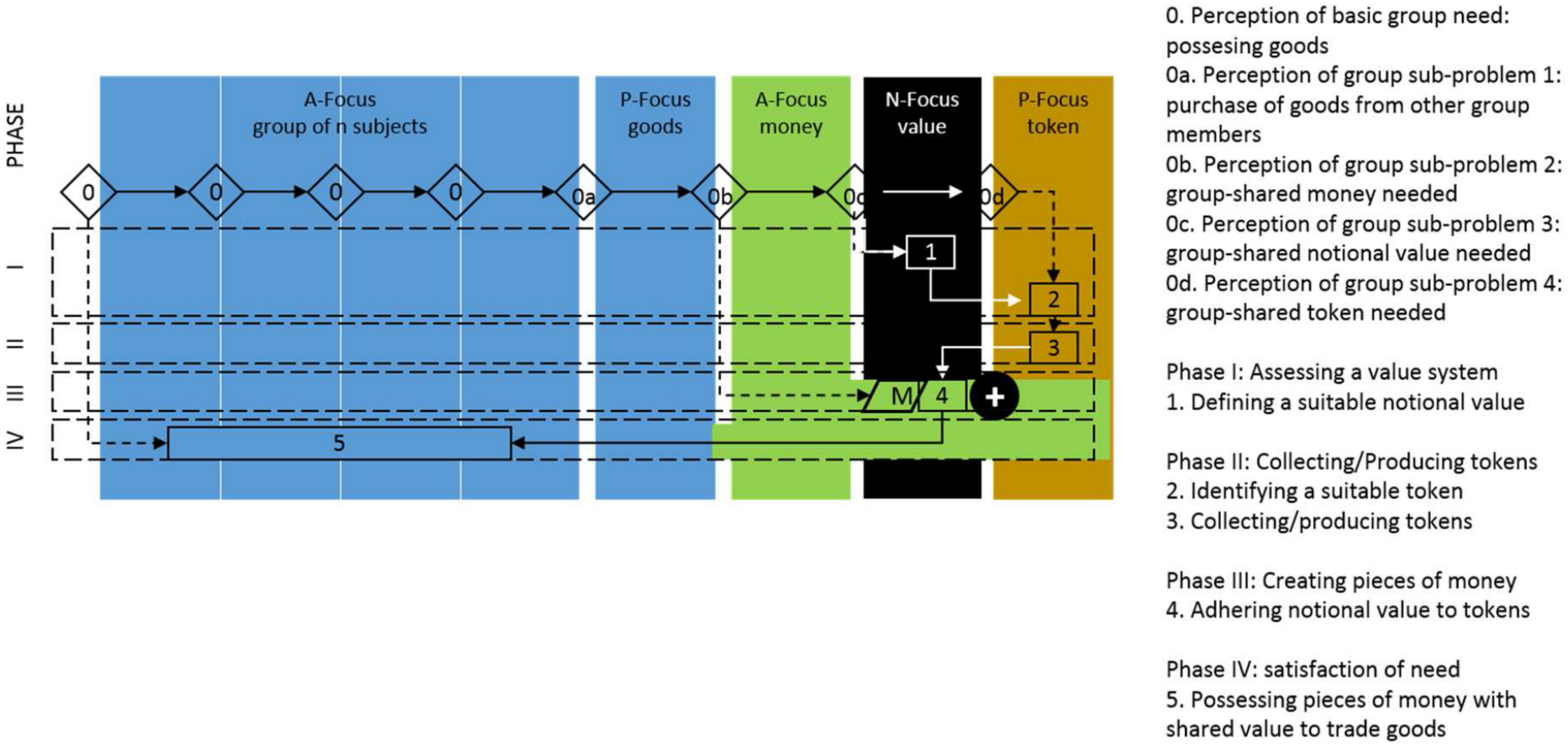
**Simplified cognigram of the creation of a piece of money with shared value**. Note the N-Focus of the notional concept of a certain value as significant fused (plus sign) with a specific token to become a new attention focus “piece of money.”

**Figure 13:**
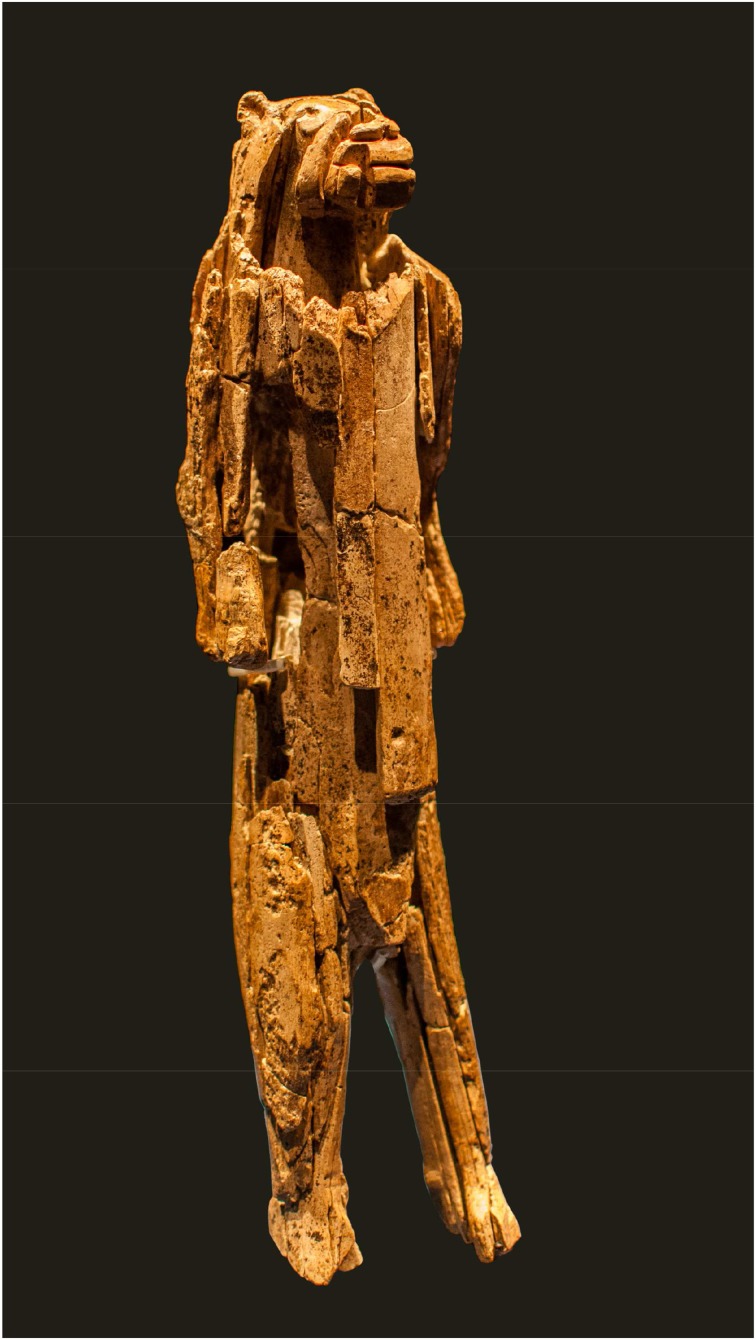
**The ivory figurine of the lion-man from Hohlenstein-Stadel, probably representing a virtual being (Photo: Dagmar Hollmann, Wikimedia Commons)**.

Finally, with notional concepts causal reasoning beyond purely physical effects of exclusively physical agents/*agens* has been introduced. As notional concepts “objects” are defined, which can be manipulated only in the mind or through imagination, but can be combined with and may have effects on physical or other notional modules. Notional concepts can be represented in (a) the signification of objects/signs (e.g., the meaning of the cross, a crescent, and the Star of David as symbols of religions), (b) systems of ideas (e.g., myths, religious beliefs, philosophical question, constitutions of states) (c) normative definitions (e.g., metric and value systems), or (d) virtual beings (e.g., angels), and characters (e.g., protecting capacities of an amulet). A notional concept as attention focus can be combined with a physical object to form a composite with new functional qualities emerging out of the basic physical qualities and a certain meaning. For example, a certain signification derived from the European monetary system can be combined with a specific metal object as token to form a coin with the economic value of 1 €. The value, however, is neither bound to the material value of the metal object nor to a specific merchandise value. Currency can be overvalued or devalued; this manipulation is primarily non-physical, although in a secondary step it has influence on the physical world indeed (Figure 12). However, there are also notional concepts, which are not linked to physical objects such as significations linked to an object to form a sign/symbol, but are independent operational foci as the ideas of “justice,” “reincarnation,” or the “devil.” Of course, the idea of “justice” is triggered by human experiences in the real world, but it is an abstract notion that can be discussed in philosophical disputes without referring to physical manifestations. Due to their nature, the detection of notional concepts or mental representations within the archeological record is difficult. If not explicitly described in written historical sources, notional concepts can only be vaguely traced from the context or tools with which they have formed composites or complementary sets. The best material expressions of notional behavioral capacities are unambiguous information carriers associated with the notional component like notations detailed and numerous enough to identify the underlying system, as for example alphabetical letters, Roman or Arabic numerical signs, or Incan quipus, a recording system using knots in sets of strings. In these cases, the depicted signs such as the letter X or the numeral 4 are physical components of a composite tool, which receives its individual qualities in combination with a mental notion. Early evidence of notional concepts are artistic representations of probably metaphysical beings such as the ca. 32,000-year-old lion-man from the Hohlestein-Stadel cave in South Germany (Figure 13) (cf. Wynn et al., 2009). For other artistic artifacts such as the ivory figurines from caves of the Swabian Jura (Conard, 2009) or parietal art in France (Vialou, 1987; Clottes, 2001), be it figurative, abstract or ornamental, a notional component is often assumed, but cannot be proven (cf. Malafouris, 2007).

### Expansion of problem-solution distances and of cultural capacities

The expansion of the problem-solution distance regarding agents and effects as described above is associated with an expansion of cultural capacities in human evolution. Cultural behavior is a subset of behavior in general, defined by a historical-social dimension of development additional to the biological and individual dimensions more or less active also in other forms of behavior (Haidle and Conard, 2011; Haidle et al., under review). Advanced tool behavior with an extended problem-solution distance is commonly not invented individually again and again, but at least some information is passively provided or actively handed down (historical aspect) by other, though not necessarily cognate members of the group (social aspect). Regarding the limited time for learning in an individual life span, the possibility to adopt knowledge and practices from other individuals becomes more important, as the problem-solution distances in single tool behavior become more complex and more different tools are used in various spheres of life. Associated with the expansion of the problem-solution distance in human evolution, the impact of the historical-social dimension to the development of (tool) behavior increases regarding the transmission of information, but also concerning the scope of application. Artifacts with notional aspects unfold their full potential only if they are used within a group that shares that notion.

**Figure 14:**
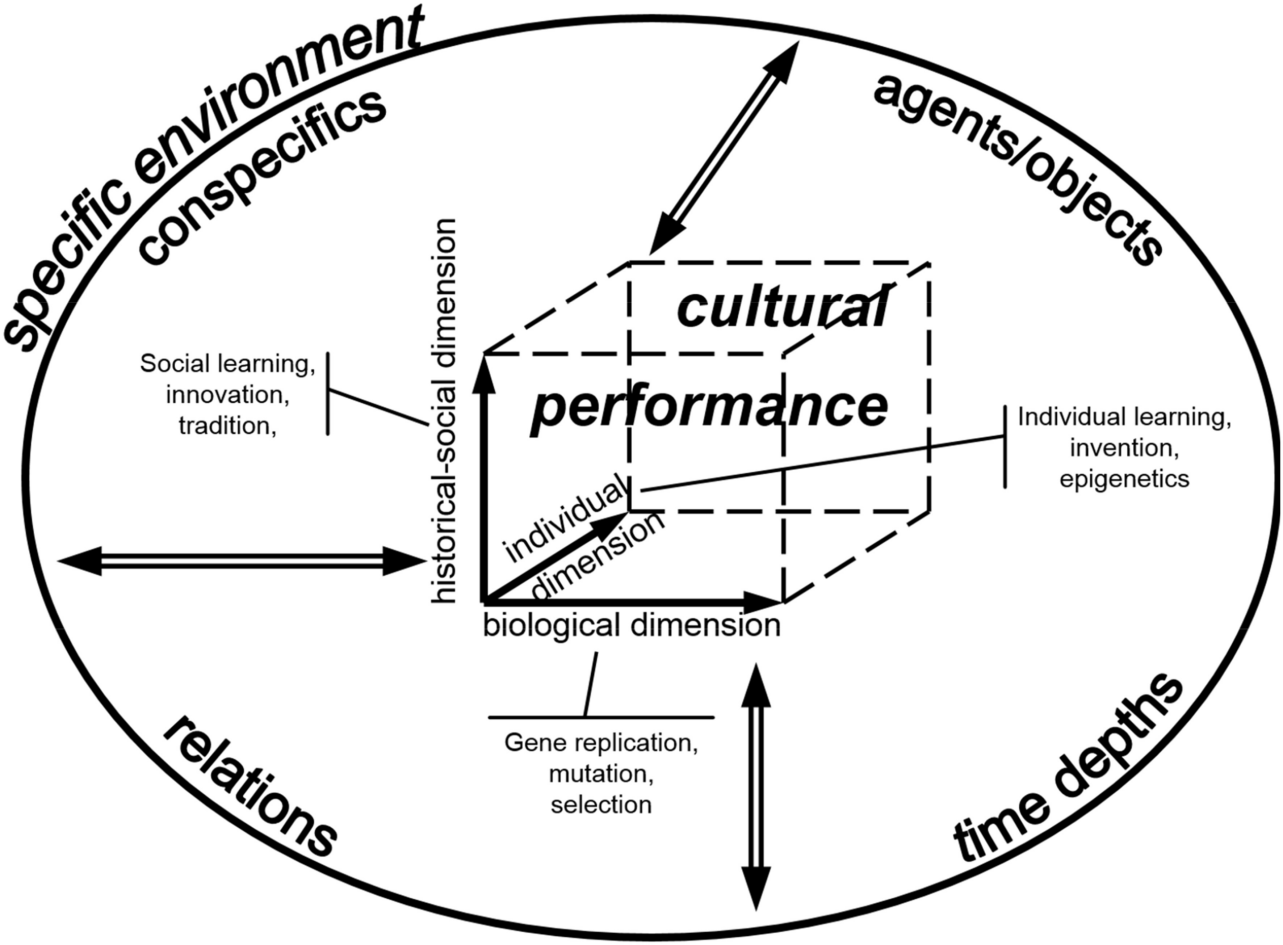
**The three dimensions of cultural performances (biological, historical-social, and individual with attached developmental processes) embedded in and interdependent (⇔) with the specific environment (from Haidle and Conard, 2011)**.

The different tool behaviors in hominins, and with it the handling of agents/*agens* and effects to satisfy individual needs, can generally be taken as different cultural performances with interrelated biological, individual and historical-social aspects of development embedded within a specific environment/resource space (Haidle and Conard, 2011; Haidle et al., under review) (Figure 14). The *biological dimension* refers to the biological potential and constraints for behavior given in genes, gene expressions, anatomical blueprints and physiological standards of a group of organisms and is expressed, for example, in the structure of the nervous system and the brain, in sensory perception, in motor and articulation skills, in the form of sociality, and in the principle abilities to communicate. The *individual dimension* of behavior reflects individuals' preferences, aversions, skills, and disabilities. The individual dimension incorporates the potential and constraints of an individual, or of a group of individuals, set by individual talents or poor aptitudes, by the personal social setting and by individual life histories of physical, mental, and emotional experiences. The *historical-social dimension* represents historical and social potentials and constraints. The set of historically acquired knowledge and skills, customs, views and opinions, and the social access to it, makes up a part of the individual's environment that can be acted on, and used as a basis for further innovation. The forms and extent of storage, transmission, permutation, and transformation of the knowledge and skills, customs, views, and opinions support or hamper the unfolding of cultural performances. The three dimensions are multifactorial and interdependent with each other and the embedding environment. This specific environment comprises conspecifics and other agents/*agens* and objects. The conspecifics, agents and objects are linked to the organisms in focus by functional relations effective within a certain time depth. The analysis of the developmental aspects of a specific behavior is thus difficult, and the identification of some factors should not entail the conclusion that all factors are understood. The same is likely to be true also for the cognitive background of the behavioral performances.

## Building the bridge—snag list

Numerous micro-theories helped to build parallel bridges from prehistoric finds to the archeological reconstructions of the activities, knowledge, and conceptions behind the manufacture and use of the tools (A–B–C–D–E). They can be set in chronological order and viewed from a problem-solution distance perspective to get an impression of the development of the handling of agents and effects in tool behavior in the course of human evolution. The final bridge arches that connect the archeological reconstructions with their possible causal-cognitive background (E–F–G–H–I) are still only in the project phase. Two main factors hamper the construction progress.

- Interpretation of the reconstructions: The coding of tool behavior in cognigrams/effective chains provides a breakdown of involved agents and their summarized effects and illustrates the implicit causal structure of a certain behavior. Yet, controlled laboratory experiments with non-human primates and different species of crows show the difficulties of determining (a) which features of an agent are perceived to cause the effect, (b) the understanding of how causes produce their effects (based on which physical mechanisms), and (c) which cognitive processes are active (e.g., Limongelli et al., 1995; Bird and Emery, 2009; Emery and Clayton, 2009; Taylor et al., 2012; for an overview see Penn and Povinelli, 2007, pp. 107–111). If it is difficult to assess to which extent a capuchin monkey or a chimpanzee understands the causal role of different features of a tool, the more this is true for the behavior/cognition of extinct hominin species. To prevent possible over-interpretation of the data, minimal explanations have to be looked for. Instead of awarding non-human primates with the capacity “to distinguish causally relevant from causally irrelevant properties of a tool and thus possess a ‘functional concept of artifacts”’ (Penn and Povinelli, 2007, p. 107), Penn and Povinelli, for example, present “a more modest hypothesis; i.e., non-human primates are predisposed to perceive certain clusters of features as more salient than others when selecting among potential tools without understanding anything about the underlying causal mechanisms involved” (Penn and Povinelli, 2007, p. 108).
- Cognitive theory: from an archeologist's perspective, a comprehensive and discrete psychological model about causal reasoning and its development seems to be lacking so far, and the neural mechanisms specifically supporting causal reasoning are poorly understood (cf. Penn and Povinelli, 2007; Osiurak et al., 2010; Vaesen, 2012, pp. 204–206). Although marked progress has been made in the last years in the study of neural mechanisms related to tool behavior in *Homo sapiens* such as the functional reorganization of visuotactile limb representations (Maravita and Iriki, 2004), the role and development of specific sectors of the parietal (Goldenberg and Spatt, 2009; Peeters et al., 2009; Bruner, 2010), and functionally specialized networks involving temporal, parietal and frontal areas within the left cerebral hemisphere (Johnson-Frey, 2004), “to date there are remarkably little data concerning the neural bases of processes required to understand physical causality of the sort necessary for complex tool use” (Johnson-Frey, 2003, p. 203). Thus, the final bridging (E–F–G–H–I) for causal reasoning can only be the fragile attempt of a temporary bridge until more stable construction elements are provided from the side of cognitive sciences. A good example of the potential of a successful bridging from archeological evidence to cognitive models is the Extended Working Memory hypothesis (Wynn and Coolidge, 2011).

## Building the bridge—summary of the project

Tool use, in most cases a cultural behavior, is commonly associated with aspects of causal cognition at least in the simplest form of understanding a causal structure. Furthermore, this understanding of an agent-effect relationship is not only retrospective, but also prospective in its application on new tasks. The manufacture of a tool fitting to a specific task additionally requires an identification of certain qualities of the tool to be gained by the modification in order to solve the basic problem (Hunt et al., 2006). Whether all necessary and sufficient qualities of the tool within the specific task are completely understood is not important; the modification of certain characteristics implies a basic causal reasoning. The basic cognitive faculties are not specific adaptations for tool behavior but domain-general cognitive capacities as experiments with rooks show, a bird species that does not use tools in the wild but appears to possess an understanding of tools (Bird and Emery, 2009). However, experiments with chimpanzees demonstrate special cognitive affordances of tool use that may obscure causal cognitive efforts. Variations of the trap-tube problem with and without tools show that “even a simple tool-using task is likely to place a load on the attentional system, because unlike the automatic movements of the hands, manipulating a tool to bring about an effective action will require increased attention. The amount needed is likely to depend on the complexity of the task, and the degree of familiarity with the tool-using action required. Moreover, the need to split attentional focus between the end of the tool that is held by the chimpanzee, the end that contacts the food, and any relevant features of the substrate on or in which the food rests (such as a trap) may be a further challenge” (Seed et al., 2009, p. 33).

The examination of the problem-solution distance with the help of cognigrams and effective chains allow us to reconstruct the causal structures in tool behavior and provides starting points for bridging the gap to the identification of (causal) cognitive capacities underlying different forms of tool behavior. *Simple tool behavior* in general requires at least minimal forms of inhibition, allowing a shift of the focus from the desired goal to a means to reach the target. The means are not chosen completely arbitrarily, but selected for a set of (necessary and random) features providing an approach to achieve the aim. The manufacture of tools is commonly directed to improve the tool's quality to help to satisfy the need. *Modular tool behavior* based on secondary tool use requires an understanding and application of causal chains. While 15-month-old children are able to understand causal chains (Cohen et al., 1999), capuchin monkeys e.g., understand only spatial relationships between two, but not three items (Fujita et al., 2003). It can be hypothesized that such a constraint is also active in chimpanzees, the most proficient tool users beside humans, which show the conception and use of sophisticated tool sets applied one after the other to the same target, but no chaining of a tool to produce another tool to achieve an aim which seems to be exclusive to hominins. The individual case of the bonobo Kanzi (Schick et al., 1999) who learned to produce flake tools with a hammerstone may simply show how years of training skills acquired in a historical-social setting from experienced individuals (here humans) can help to overcome cognitive limits. *Composite tool behavior* also requires the combination of different tools with different qualities. Instead of being applied in a causal sequence, however, the tools with different qualities joint in a composite tool unfold their effects together and interdependently to reach the target. In modular as well as in composite tool behavior the subject triggers the application of each tool in a sequence independently. In *complementary tool behavior*, in contrast, only the controlling part of the tool set is activated, which then gives an impulse on the other part of the tool set in order to achieve the desired aim. To conceptualize and produce a functional complementary tool set the application of a subject-independent trigger extends the necessary causal understanding. *Notional tool behavior*, finally, requires abstract causal reasoning about not observable agents and their effects. Though also New Caledonian crows reason about hidden causal agents (Taylor et al., 2012), it is unclear what the animals expect to be the causal agent. In notional tool behavior the subject does not only look for hidden causal agents, but mental representations as tools respectively, components of tools are applied. Penn and Povinelli (2007, p. 111) emphasize a strong relationship of abstract causal reasoning with analogical reasoning. Vaesen (2012, p. 266) summarizes that “causal thought involves both the ability to infer causal mechanisms relating cause-effect covariances (i.e., inferential causal reasoning) and the ability to recognize that such mechanisms underpin causally analogous events (i.e., analogical causal reasoning). Current evidence suggests that chimpanzees perform rather modestly in both respects. Humans, in contrast, have a drive for seeking and generalizing causal explanations, and often learn about causality through their own diagnostic interventions—a behavior not yet observed in the great apes.” Additionally, *Homo sapiens* is able to conceptualize mental representations as agents/*agens*.

The studies on prehistoric tool behavior presented above strongly suggest a multi-leg evolution of several components of causal cognition and adjacent cognitive features. Additionally, the development of the different cultural performances of tool behavior is not only based on biological and individual factors, but also on historical-social factors. The three dimensions are multifactorial, interdependent, and embedded in the specific environment of the population (Haidle and Conard, 2011). The same can be assumed for the different performances in the cognitive sphere. A cultural performance may have different cognitive backgrounds. Prior individual experience helps to manage a new task (von Bayern et al., 2009); historical-socially transmitted experience of other individuals in cultural context can do the same. If trained by a knowledgeable individual, naïve individuals may perform very well in a lot of problem settings also with sophisticated tools, and without understanding the basic causal relations. Consequently, not all individuals in a group with cultural behavior have to share the same cognitive capacities to perform in some aspects in the same cultural way. And with the same cultural capacities of problem-solving different individuals and populations may perform very differently. I assume that the cultural background, respectively the historical-social dimension also shapes the cognitive performances behind the behavior. There are no data available about past human cognition. But with the help of argumentative bridges at least some impressions on the evolution of causal cognition can be gained from prehistoric artifacts.

### Conflict of interest statement

The author declares that the research was conducted in the absence of any commercial or financial relationships that could be construed as a potential conflict of interest.
